# Effects of Personalized Nutrition Education on Lipid Profiles in Chinese Adults: A Medical Student-Implemented Community Intervention Study

**DOI:** 10.3390/nu17132161

**Published:** 2025-06-28

**Authors:** Hongli Wang, Tianyi Shen, Jingming Zhu, Jing Gao, Shaoxian Liang, Wanshui Yang, Zhuang Zhang

**Affiliations:** Department of Nutrition and Hygiene, School of Public Health, Anhui Medical University, Hefei 230032, China; wanghongli132000@163.com (H.W.); shentianyi624@163.com (T.S.); zhujingming956@163.com (J.Z.); m17821035817@163.com (J.G.); liangshaoxian98@163.com (S.L.)

**Keywords:** nutrition education, blood lipid profiles, dyslipidemia

## Abstract

Background: Dyslipidemia affects over 30% of Chinese adults, with awareness rates below 20%. Promoting nutrition education programs in the general population is important, but few studies have investigated the specific strategies and their efficacy. Methods: This longitudinal study was conducted in a representative sample of community-dwelling adults in Lu’an City, central-eastern China. After recruitment and propensity score matching, the personalized and conventional nutrition education groups included 306 and 612 participants, respectively. We provided standardized nutrition education based on the Dietary Guidelines for Chinese Residents (2022) for the conventional group, and personalized nutrition advice based on individual food intake and the guidelines for the personalized group. Serum total cholesterol (TC), high-density lipo-protein cholesterol (HDL-C), low-density lipoprotein cholesterol (LDL-C) and triglycerides (TGs) were measured at baseline and again after follow-up. Results: Three years after the intervention, the average levels of TC, LDL-C, and TG in both groups all increased. After adjusting for covariates, an increase in HDL-C was greater in the personalized group than in the convention group, while the increment in TG was less pronounced. LDL-C changes were similar between the groups. The beneficial effect of personalized nutrition education on HDL-C levels was more pronounced in women (*p*_interaction_ < 0.05). Similar results were observed among participants with dyslipidemias and after excluding all lipid-lowering medication users. Conclusions: We conducted personalized nutrition education through medical students’ community practice projects among Chinese community residents, revealing that personalized nutrition education based on dietary surveys could significantly improve blood lipid profiles in general residents and individuals with dyslipidemias compared to conventional nutrition education.

## 1. Introduction

Dyslipidemias are defined as raised plasma concentrations of total cholesterol (TC), low-density lipoprotein cholesterol (LDL-C) or triglycerides (TGs), a low plasma concentration of high-density lipoprotein cholesterol (HDL-C), or a combination of these features. The global burden of dyslipidemias has increased over the past 30 years [1], especially in developing countries, including China, as a consequence of dietary and behavioral changes [2]. Dyslipidemias are major risk factors for cardiovascular diseases (CVDs), which are the leading cause of death worldwide [3]. Each 1 mmol/L reduction in LDL-C could prevent 11 per 1000 major vascular events over 5 years [4]. Well-documented evidence suggests the benefits of healthy dietary patterns on the prevention and management of dyslipidemia [5,6,7]. Medical nutrition therapy embedded with nutrition education has been shown to improve metabolic profiles in patients with dyslipidemia, even in the absence of lipid-lowering medications [8,9].

However, many patients are not aware of their conditions, especially in developing countries [10]. Dyslipidemia affects over 30% of Chinese adults, with awareness rates below 20% [11,12]. Therefore, promoting nutrition education programs for the general population is important to improve the lipid profiles of Chinese residents [10]. Despite the established role of nutrition education in clinical settings [8,9], evidence on its effectiveness for improving lipid profiles in general community populations remains scarce, particularly in low- and middle-income countries. Moreover, optimal intervention strategies warrant further investigation. Dietary Guidelines for Chinese Residents are dietary recommendations for general residents in China and have been widely adopted in community-based nutrition education programs [13].

Personalized nutrition education has been reported to be more beneficial than conventional nutrition education to improve the health status [14,15]. However, in China’s community-based healthcare system, individualized nutrition education remains limited due to physicians’ nutritional knowledge gaps [16,17]. Meanwhile, universities—particularly medical schools—frequently conduct dietary survey projects targeting community residents, where interviewers are typically medical students with training in both medicine and nutrition. Utilizing these dietary survey results to provide personalized dietary guidance may simultaneously benefit residents’ nutrition and students’ skill development, without imposing too many additional burdens on the project. Nevertheless, the effectiveness of this approach in improving lipid profiles among general populations, particularly its long-term effects, remains unclear.

This study aims to investigate whether personalized nutrition education based on dietary surveys conducted through medical students’ community practice projects can effectively improve blood lipid levels in general Chinese adults.

## 2. Materials and Methods

### 2.1. Study Population

Participants in this study were selected from the Anhui Liver Disease Study (ALDS). The design and the rationale of the ALDS were described elsewhere [18,19]. Briefly, the ALDS is an ongoing prospective cohort in a representative sample of community-dwelling adults aged ≥18 years in Lu’an and Ma’anshan, China. The study protocol has been approved by the ethics committee of Anhui Medical University (protocol number: 20210730), and all participants provided written informed consent. Nutrition education intervention in this study was implemented among the 2942 participants in Lu’an City.

Baseline surveys were conducted during 2020–2021. Among all 2942 participants in the baseline, a total of 754 participants were recruited for the personalized nutrition education group, while the remaining 2188 received conventional nutrition education. We excluded participants if they (i) were lost to follow-up (*n* = 729), (ii) did not provide blood samples (*n* = 199), or (iii) had missing data on covariates (*n* = 96). After exclusion, a total of 1918 participants were included, including 422 in the personalized nutrition intervention group, and 1496 in the conventional nutrition education group. Propensity score matching (matching ratio of 1:2 and a caliper of 0.2) was applied to balance sex, age, body mass index (BMI), and blood lipid levels at baseline between groups, yielding 306 personalized nutrition education recipients and 612 controls receiving conventional nutrition education.

### 2.2. Dietary Assessments

Dietary intake was assessed at baseline but not during follow-up due to constraints imposed by the study design. We used a 141-item food frequency questionnaire (FFQ), which was previously validated in the Anhui Lifestyle Validation Study (ALVS, 2021–2022), showing reasonable reproducibility and validity for nutrient and dietary pattern assessment to collect 1-year habitual dietary information through face-to-face interviews at baseline [20]. In this study, the FFQ demonstrated good reproducibility, with 62.5% of food groups showing ICCs > 0.50. For validity against 24 h dietary recalls, 75.0% of food groups exhibited correlation coefficients exceeding the 0.20 acceptability threshold for dietary assessment tools (unpublished data) [21].

Participants reported the average consumption frequency of a standardized portion size for each food item over the past year, selecting from nine frequency categories ranging from “never” to “three times per day”. Average daily food intake was calculated by multiplying the reported consumption frequency by the specified portion size for each food item. All medical student interviewers received standardized training in food portion estimation using food photographs and measurement protocols.

### 2.3. Description of the Intervention

The nutrition education interventions were delivered at baseline. For participants in the conventional nutrition education group, we provided standardized nutrition education based on the Dietary Guidelines for Chinese Residents (2022) following FFQ completion, with trained medical student interviewers providing verbal explanations of the general recommendations to each participant in brief one-on-one sessions (3–5 min). This non-personalized education covered the following daily intake recommendations: 200–300 g of grains, 50–100 g of roots and tubers, 300–500 g of vegetables, 200–350 g of fruits, 120–200 g of animal-based foods including meat, poultry, fish, and egg products, 300–500 g of milk and dairy products, 25–35 g of pulses, nuts, and seeds, less than 5 g of salt, and no more than 25–30 g of cooking oil (Table S1).

For participants in the personalized nutrition education group, we calculated their intake of each food group based on dietary survey results and compared it with the recommended amounts in the Dietary Guidelines for Chinese Residents (2022). For food groups, where intake fell below the guidelines, we advised increasing consumption; where intake exceeded the guidelines, we suggested reducing consumption. For example, when a participant’s milk and dairy products intake was 200 g (below the 300–500 g recommendation), the report displayed “Milk and dairy products: 200 g (below recommended range)”, along with specific suggestions to increase consumption. The actual intake, recommended intake, and personalized food intake advice were all documented in a printed report, which was then provided to each participant in the personalized nutrition education group.

### 2.4. Blood Markers

Participants were requested to provide fasting blood samples at baseline and again after follow-up. Serum TC, HDL-C, LDL-C, and TG were quantified by electrochemiluminescence. The TC/HDL-C ratio was also calculated by dividing the TC level by the HDL-C level as an integrated indicator of the overall lipid-related cardiovascular risk [22]. Dyslipidemia was defined if any of the following criteria were met: hypercholesterolemia (TC ≥ 5.2 mmol/L), hypertriglyceridemia (TG ≥ 1.7 mmol/L), high LDL-C (LDL-C ≥ 3.4 mmol/L), or low HDL-C (HDL-C < 1.0 mmol/L) [23,24]. Printed lipid test reports were distributed to all participants in both groups.

### 2.5. Assessment of Covariates

Information on age, gender, education level, smoking status, alcohol drinking, physical activity, annual household income, and disease history was collected using a self-reported questionnaire. The body weight and height were measured by a professional investigator. The BMI was calculated by dividing body weight (kg) by squared height (m^2^). Physical activity was quantified as metabolic equivalent tasks (METs)-h/week. Total energy intake was calculated according to the FFQ.

### 2.6. Statistical Analysis

The characteristics of the participants were presented as means with standard deviation (SD) for continuous variables and percentages for categorical variables. Serum lipid level changes were calculated by subtracting baseline lipid levels from follow-up lipid levels. Differences in lipid changes between the two groups were compared using an analysis of covariance (ANCOVA). Multivariable-adjusted linear regression models were used to assess the effects of personalized nutrition education on the serum profile changes compared with conventional nutrition education. In all ANCOVA and linear regression analyses, adjustments were made for age (years), sex (female, male), education level (uneducated, primary school or below, junior high school, and college or above), annual household per capita income (<5000 yuan, 5000–10,000 yuan, 10,000–20,000 yuan, ≥20,000 yuan), marital status (married and others), BMI (kg/m^2^), smoking status (never, former, current smokers), alcohol drinking (never, former, current drinkers), physical activity (METs-h/week), and total energy intake (kcal/day) at baseline.

Subgroup analyses were conducted to examine potential effect modifications by selected covariates, including age (18–39 years, 40–59 years, and ≥60 years), sex (male and female), and education level (primary school or below and junior high school or above). To evaluate the effects of personalized nutrition education in dyslipidemia patients, we conducted ANCOVA and linear regression analyses among participants with dyslipidemia. Sensitivity analyses were performed after excluding all lipid-lowering medication users to control for potential confounding.

All statistical tests were two-sided, with a significance level set at *p* < 0.05. Analyses were conducted using R (version 4.4.2).

## 3. Results

### 3.1. Participant Characteristics

This study included 918 participants (mean age 52 years), with 612 in the conventional nutrition education group and 306 in the personalized nutrition education group. After propensity score matching, baseline characteristics were generally balanced between the two groups, though the personalized group had a higher proportion of individuals with an annual household income (*p* = 0.01). No significant differences were observed in age, sex, marital status, education level, smoking status, BMI, physical activity, and lipid profiles (all *p* > 0.05; Table 1).

In the personalized nutrition education group, compliance with the Dietary Guidelines for Chinese Residents (2022) is presented in Table S1. Notably, 95.7% of participants consumed less milk and dairy products than the recommended intake, 87.1% fell short in roots and tubers, and 78.0% had inadequate fruit intake. Excessive intake was prevalent for oils and salt. Only a minority met recommendations for cereals, vegetables, and meat/poultry/fish (Table S2).

### 3.2. Changes in Lipid Profiles Following Nutrition Education

Three years after the intervention, the average levels of TC, LDL-C, and TG in both groups all increased. The average level of HDL-C of the conventional nutrition education group decreased by 0.01 mmol/L (0.83%), but that of the personalized nutrition education group increased by 0.11 mmol/L (9.17%). The TC levels of the personalized nutrition education group increased more than the conventional nutrition education group (*p* = 0.04). The personalized group exhibited a more modest rise in TG levels relative to the control group (*p* = 0.03). LDL-C changes were similar between groups. The TC/HDL-C ratio increased more in the conventional group than in the personalized group (*p* < 0.01, Table 2). Lipid profile changes in mg/dL units are presented in Table S3.

Among participants with dyslipidemia, similar results were observed. Personalized nutrition education outperformed conventional nutrition education, with greater HDL-C improvements, attenuated TG elevation, and less rise in the TC/HDL-C ratio (*p* = 0.04, Table S4).

### 3.3. Effects of Personalized Nutrition Education on Serum Lipid Profiles

The results of linear regression analyses revealed that personalized nutrition education significantly improved lipid profiles compared to conventional education. After adjusting for possible confounders, TC and HDL-C levels rose 0.14 (95% CI: 0.01 to 0.27) and 0.12 (95% CI: 0.09 to 0.16) mmol/L more versus the conventional group, respectively. The TG increase in the intervention group was 0.21 (95% CI: −0.02 to −0.39) mmol/L less versus the conventional group, and the TC/HDL-C ratio in the intervention group increased by 0.31 (95% CI: −0.45 to −0.17) less versus the conventional group. No significant difference in LDL-C change was observed (Table 3). Similar trends were observed in adults with dyslipidemia (Table 4) and those not using lipid-lowering drugs (*n* = 891) (Table S5).

### 3.4. Subgroup Analyses of Nutrition Education Effects on Lipid Profile Changes

Subgroup analyses were performed to assess the observed beneficial effects of personalized nutrition education on HDL-C and TG based on sex, age, and education level. The beneficial effect of HDL-C was more pronounced among women [β (95% CI) = 0.18 (0.14, 0.22) mmol/L] but was attenuated among men (*p*_interaction_ < 0.01, Table 5). For TG and TC/HDL-C ratio changes, no significant interaction effect was observed (Tables S6 and S7).

## 4. Discussion

In this community-based longitudinal study, participants receiving personalized nutrition education based on dietary surveys showed more favorable changes in blood lipid profiles over three years compared to those receiving conventional nutrition education. These improvements were more evident among women.

Previous research investigating the effects of nutrition education on blood lipids has mainly been conducted in patients, with very few studies involving healthy populations—most of these focused on students and adolescents [25,26,27]. One intervention study targeting urban Asian Indian adolescents that included personalized nutritional counseling observed a significant decrease in TG levels in the intervention group [25]. Similarly, a longitudinal randomized trial found that repeated individualized dietary counseling significantly reduced the risk of high TG in male adolescents [26], consistent with our results. In addition to TG changes, our study further identified that the intervention group exhibited a greater increase in HDL-C levels compared to the control group. The improvement in HDL-C was accompanied by favorable changes in the TC/HDL-C ratio, a composite indicator of cardiovascular risk that reflects both atherogenic and protective lipid components [22]. We also observed a greater TC increase in the personalized nutrition education group. Results in Table 3 suggested that the difference in TC increase (β [95% CI]: 0.14 [0.01, 0.27]) was primarily attributable to the difference in HDL-C increase (β [95% CI]: 0.12 [0.09, 0.16]). However, another non-personalized 12-week integrated program of gardening, nutrition, and cooking activities for predominantly Hispanic/Latino youth from low-income backgrounds showed no significant differences in lipid changes between groups [27]. Substantial evidence indicates that personalized nutrition education yields better outcomes than non-personalized approaches [14,15], which is supported by the present study. Our study participants were community residents, predominantly with less than 9 years of formal education and limited nutrition knowledge. Conventional dietary education proved less effective because participants lacked awareness of their actual food consumption relative to the recommended amounts. In comparison, personalized nutrition education derived from dietary surveys provided concrete, specific guidance on appropriate consumption quantities for each food category. Our findings suggested that for the general population, personalized nutrition education based on dietary surveys may yield better outcomes.

This study also found that personalized nutrition advice improved blood lipid profiles in individuals with dyslipidemia. Previous studies on nutrition education for dyslipidemia patients have consistently reported improvements in blood lipids [28,29,30]. A nutrition intervention study based on the BASNEF model in 150 people with a high blood lipid profile found that after two months, TG, TC, and LDL levels decreased significantly in the intervention group, while the HDL level increased significantly after the intervention [28]. A randomized controlled field trial in 104 employees with dyslipidemia from a regional petrochemical company found that tailored nutrition education significantly reduced LDL-C [29]. Another study using a digital nutrition platform for nutrition intervention in 653 dyslipidemia patients found that TC and LDL-C decreased significantly, and HDL-C increased significantly in the intervention group [30]. In the present study, three years after our intervention, TG increased less in the intervention group than in the control group, consistent with previous studies. However, we did not find a difference in LDL-C changes between the two groups, in the general population, or in individuals with dyslipidemia. This discrepancy in the LDL-C response may be attributed to individual biological diversity, including variations in gut microbiota composition and metabolic enzyme activities that influence lipid absorption and metabolism [31]. Another possible reason includes the fact that our study population consisted of community-dwelling general residents, and even those with dyslipidemia had better baseline lipid profiles—especially LDL-C (mean: 105 mg/dL)—compared to dyslipidemia patients in the aforementioned studies. Additionally, different dietary habits may have played a role. Since we did not conduct comprehensive dietary surveys during follow-up, we were unable to assess actual dietary changes. However, baseline dietary data showed that the study population’s intake of legumes, nuts, tubers, and fruits was significantly lower than the recommended levels. These foods are rich in soy protein, soluble fiber, and monounsaturated fatty acids, all of which contribute to LDL-C reduction [32,33,34]. In addition, it is also possible that the limited intervention intensity, as we only delivered the nutrition education at baseline without subsequent reinforcement, may have been insufficient to produce sustained effects on LDL-C levels.

The subgroup analysis revealed that the nutritional intervention yielded better outcomes in women. Previous studies have also indicated that women tend to pay greater attention to nutrition, diet, and health, making them more likely to modify their behaviors in response to dietary recommendations and thus achieve greater benefits [35,36]. Additionally, women have a more favorable lipid profile compared to men, largely due to the effects of endogenous estrogen [37]. Prior research has suggested that education level may influence the knowledge, attitudes, and practices related to nutrition education [38,39,40]. However, this study did not observe a significant impact of education level on the effectiveness of the intervention. Although improvements in HDL-C were more pronounced in participants with primary school education or below compared to those with secondary education or above, no significant interaction effect was observed. This may be attributed to the overall low education level of the study population.

The strengths of this study lie in comparing the differences between personalized nutrition advice based on dietary surveys and non-personalized nutrition education in the general population, thereby providing a basis for formulating nutrition education strategies for the general public. Secondly, this study added dietary recommendations to existing university research projects, exploring a nutrition education approach that neither imposes an excessive burden on the research nor fails to improve residents’ health. Finally, the three-year follow-up period enabled us to observe long-term changes in lipid profiles. This study conducted a three-year follow-up, which can indicate the long-term effects of the interventions. However, the limitations of this study include the lack of randomized grouping of the study subjects. Although propensity score matching was used for controls, there may still be confounding factors. Additionally, the absence of specific dietary intake assessments at follow-up limits interpretations regarding adherence to dietary recommendations and actual behavioral changes in this study. The lack of nutrition knowledge assessment precluded further investigation into the reason behind the blood lipid modifications.

## 5. Conclusions

In this study, we conducted personalized nutrition education through medical students’ community practice projects among Chinese community residents. It was found that this nutrition education approach could potentially lead to improvements in long-term blood lipid profiles in both general residents and patients with dyslipidemia. This study explored a new possibility for nutrition education targeting the general Chinese population: combining nutrition survey projects from medical students’ community practice projects with community nutrition education may be an economical and effective strategy.

## Figures and Tables

**Table 1:** Baseline characteristics of participants after propensity score matching.

Characteristics	Conventional Nutrition Education Group	Personalized Nutrition Education Group	*p*
No. of participants	612	306	
Age, years	51.86 (14.76)	52.25 (14.58)	0.70
Sex, %			0.29
Male	280 (45.75)	128 (41.83)	
Female	332 (54.25)	178 (58.17)	
Annual household per capita income, % ^1^			0.01
<5000 yuan	109 (17.81)	65 (21.24)	
5000–10,000 yuan	171 (27.94)	57 (18.63)	
10,000–20,000 yuan	158 (25.82)	74 (24.18)	
≥20,000 yuan	174 (28.43)	110 (35.95)	
Marital status, %			0.16
Married	538 (87.91)	258 (84.31)	
Others	74 (12.09)	48 (15.69)	
Education, %			0.27
Primary school or below	347 (56.70)	161 (52.61)	
Junior high school or above	265 (43.30)	145 (47.39)	
Smoking status, %			0.30
Never smokers	429 (70.10)	225 (73.53)	
Former smokers	58 (9.48)	20 (6.54)	
Current smokers	125 (20.42)	61 (19.93)	
Alcohol drinking, %			0.05
Never drinkers	464 (75.82)	236 (77.12)	
Former drinkers	49 (8.01)	12 (3.92)	
Current drinkers	99 (16.18)	58 (18.96)	
BMI, kg/m^2^	24.53 (3.47)	24.64 (3.57)	0.67
Total energy intake, kcal/day	2859.11 (1170.88)	2867.04 (1017.09)	0.92
Physical activities, METS-h/week	170.71 (114.10)	169.15 (106.29)	0.84
TC, mmol/L	4.47 (0.92)	4.44 (0.92)	0.66
HDL-C, mmol/L	1.21 (0.25)	1.20 (0.36)	0.81
LDL-C, mmol/L	2.65 (0.73)	2.63 (0.67)	0.62
TG, mmol/L	1.45 (1.19)	1.54 (1.33)	0.28
TC/HDL-C ratio	3.77 (0.71)	3.78 (0.66)	0.83

BMI, body mass index; METS, metabolic equivalent task; TC, total cholesterol; HDL-C, high-density lipoprotein cholesterol; LDL-C, low-density lipoprotein cholesterol; TG, triglyceride. Continuous variables are expressed as the mean (SD) according to the distribution of the variables, while categorical variables are presented as %. ^1^ Currency conversion: 1 Chinese yuan ≈ 0.14 US dollars (based on the average exchange rate during the study period).

**Table 2:** Changes in lipid profiles before and after nutrition education within each group.

Lipid Profiles	Conventional Nutrition Education Group			Personalized Nutrition Education Group			*p*
	Baseline	Follow-Up	Change	Baseline	Follow-Up	Change	
TC, mmol/L	4.47 ± 0.92	5.09 ± 1.08	0.62 ± 0.85	4.44 ± 0.92	5.19 ± 1.28	0.75 ± 1.08	0.04
HDL-C, mmol/L	1.21 ± 0.25	1.20 ± 0.27	−0.01 ± 0.22	1.20 ± 0.36	1.31 ± 0.29	0.11 ± 0.36	<0.01
LDL-C, mmol/L	2.65 ± 0.73	3.03 ± 0.75	0.37 ± 0.64	2.63 ± 0.67	3.02 ± 0.77	0.39 ± 0.63	0.72
TG, mmol/L	1.45 ± 1.19	1.73 ± 1.30	0.28 ± 1.21	1.54 ± 1.33	1.64 ± 1.77	0.10 ± 1.54	0.03
TC/HDL-C ratio	3.77 ± 0.71	4.39 ± 1.10	0.62 ± 0.85	3.78 ± 0.66	4.10 ± 1.32	0.32 ± 1.24	<0.01

TC, total cholesterol; HDL-C, high-density lipoprotein cholesterol; LDL-C, low-density lipoprotein cholesterol; TG, triglyceride. Data are presented as mean ± SD. *p* values were calculated by ANCOVA to compare the changes in the serum lipid levels between groups, adjusting for age (years), sex (female and male), education level (primary school or below and junior high school or above), annual household per capita income (<5000 yuan, 5000–10,000 yuan, 10,000–20,000 yuan, ≥20,000 yuan), marital status (married and others), BMI (kg/m^2^), smoking status (never, former, current smokers), alcohol drinking (never, former, current drinkers), physical activity (METs-h/week), and total energy intake (kcal/day) at baseline.

**Table 3:** Effects of personalized nutrition education on the serum profile changes compared with conventional nutrition education in all participants.

Lipid Profile Changes	β (95% CI)		*p*
	Conventional Nutrition Education Group	Personalized Nutrition Education Group	
TC, mmol/L	Reference	0.14 (0.01, 0.27)	0.04
HDL-C, mmol/L	Reference	0.12 (0.09, 0.16)	<0.01
LDL-C, mmol/L	Reference	0.02 (−0.07, 0.10)	0.72
TG, mmol/L	Reference	−0.21 (−0.39, −0.02)	0.03
TC/HDL-C ratio	Reference	−0.31 (−0.45, −0.17)	<0.01

TC, total cholesterol; HDL-C, high-density lipoprotein cholesterol; LDL-C, low-density lipoprotein cholesterol; TG, triglyceride. *p* values were calculated in linear regression models to assess the difference in serum profile changes in the personalized nutrition education group compared with the conventional nutrition education group, adjusting for age (years), sex (female and male), education level (primary school or below and junior high school or above), annual household per capita income (<5000 yuan, 5000–10,000 yuan, 10,000–20,000 yuan, ≥20,000 yuan), marital status (married and others), BMI (kg/m^2^), smoking status (never, former, current smokers), alcohol drinking (never, former, current drinkers), physical activity (METs-h/week), and total energy intake (kcal/day) at baseline.

**Table 4:** Effects of personalized nutrition education on the serum profile changes compared with conventional nutrition education in participants with dyslipidemia.

Lipid Profile Changes	β (95% CI)		*p*
	Conventional Nutrition Education Group	Personalized Nutrition Education Group	
TC, mmol/L	Reference	0.23 (0.02, 0.44)	0.03
HDL-C, mmol/L	Reference	0.10 (0.04, 0.16)	<0.01
LDL-C, mmol/L	Reference	0.08 (−0.06, 0.22)	0.28
TG, mmol/L	Reference	−0.38 (−0.65, −0.10)	0.01
TC/HDL-C ratio	Reference	−0.24 (−0.47, −0.01)	0.04

TC, total cholesterol; HDL-C, high-density lipoprotein cholesterol; LDL-C, low-density lipoprotein cholesterol; TG, triglyceride. *p* values were calculated in linear regression models to assess the difference in serum profile changes in the personalized nutrition education group compared with the conventional nutrition education group, adjusting for age (years), sex (female and male), education level (primary school or below and junior high school or above), annual household per capita income (<5000 yuan, 5000–10,000 yuan, 10,000–20,000 yuan, ≥20,000 yuan), marital status (married and others), BMI (kg/m^2^), smoking status (never, former, current smokers), alcohol drinking (never, former, current drinkers), physical activity (METs-h/week), and total energy intake (kcal/day) at baseline.

**Table 5:** Effects of personalized nutrition education on HDL-C changes compared with conventional nutrition education in different subgroups.

Subgroup	*n*	β (95% CI)	*p*_interaction_
Sex			<0.01
Male	408	0.05 (−0.02, 0.12)	
Female	510	0.18 (0.14, 0.22)	
Age			0.68
18–39	208	0.18 (0.11, 0.25)	
40–59	419	0.12 (0.05, 0.19)	
≥60	291	0.12 (0.07, 0.18)	
Education level			0.74
Primary school or below	508	0.14 (0.09, 0.18)	
Junior high school or above	410	0.12 (0.05, 0.18)	

HDL-C, high-density lipoprotein cholesterol. *p*_interaction_ values were calculated in linear regression models to assess the difference in HDL-C change in the personalized nutrition education group compared with the conventional nutrition education group, adjusting for age (years), sex (female and male), education level (primary school or below and junior high school or above), annual household per capita income (<5000 yuan, 5000–10,000 yuan, 10,000–20,000 yuan, ≥20,000 yuan), marital status (married and others), BMI (kg/m^2^), smoking status (never, former, current smokers), alcohol drinking (never, former, current drinkers), physical activity (METs-h/week), and total energy intake (kcal/day) at baseline.

## Data Availability

The raw data supporting the conclusions of this article will be made available by the authors on request.

## Supplementary Materials

The following supporting information can be downloaded at: https://www.mdpi.com/article/10.3390/nu17132161/s1; Table S1: Recommended food intake in the Dietary Guidelines for Chinese Residents (2022); Table S2: Compliance rates with Dietary Guidelines for Chinese Residents by food groups in the personalized nutrition education group; Table S3: Changes in lipid profiles before and after nutrition education within each group; Table S4: Changes in lipid profiles before and after nutrition education within each group among participants with dyslipidemias; Table S5: Effects of personalized nutrition education on the serum profile changes compared with conventional nutrition education after exclusion of lipid-lowering medication users; Table S6: Effects of personalized nutrition education on TG changes compared with conventional nutrition education in different subgroups; Table S7: Effects of personalized nutrition education on TC/HDL-C ratio changes compared with conventional nutrition education in different subgroups.

## Abbreviations

The following abbreviations are used in this manuscript:

TC	Total cholesterol
LDL-C	Low-density lipoprotein cholesterol
TGs	Triglycerides
HDL-C	High-density lipoprotein cholesterol
CVDs	Cardiovascular diseases
ALDS	Anhui Liver Disease Study
ALVS	Anhui Lifestyle Validation Study
ICCs	Intraclass correlation coefficients
BMI	Body mass index
FFQ	Food frequency questionnaire
METs	Metabolic equivalent tasks
SD	Standard deviation
ANCOVA	Analysis of covariance
