# Supplementary material for: Effects of Personalized Nutrition Education on Lipid Profiles in Chinese Adults: A Medical Student-Implemented Community Intervention Study
Source: Nutrients. 2025 Jun 28;17(13):2161. doi: 10.3390/nu17132161 (PMC12251181; doi:10.3390/nu17132161)
Supplement: Supplementary file 1 [file nutrients-17-02161-s001.zip › nutrients-3708811-supplementary.pdf]

Table S1. Recommended food intake in the Dietary Guidelines for Chinese Residents (2022).

Food intake	Daily intake recommendations
Grains	200-300 g
Roots and tubers	50-100 g
Vegetables	300-500 g
Fruits	200-350 g
Meat, poultry, fish, and egg products	120-200 g
Milk and dairy products	300-500g
Pulses, nuts, and seeds	25-35g
Salt	< 5g
Cooking oil	25-30g

Table S2. Compliance rates with Dietary Guidelines for Chinese Residents by food groups in the personalized nutrition education group.

Food intake	No. of participants (%)
**Grains**	
Below recommended intake	62.3
Within recommended intake	15.7
Over recommended intake	22.0
**Roots and tubers**	
Below recommended intake	87.1
Within recommended intake	9.9
Over recommended intake	3.0
**Vegetables**	
Below recommended intake	48.7
Within recommended intake	31.9
Over recommended intake	19.4
**Fruits**	
Below recommended intake	78.0
Within recommended intake	14.4
Over recommended intake	7.5
**Meat, poultry, fish, and egg products**	
Below recommended intake	50.0
Within recommended intake	32.2
Over recommended intake	17.8
**Milk and dairy products**	
Below recommended intake	95.7
Within recommended intake	3.9
Over recommended intake	0.3
**Pulses, nuts, and seeds**	
Below recommended intake	77.7
Within recommended intake	9.5
Over recommended intake	12.8
**Salt**	
Within recommended intake	20.7
Over recommended intake	79.3
**Cooking oil**	
Below recommended intake	5.6
Within recommended intake	7.3
Over recommended intake	87.0

Table S3. Changes in lipid profiles before and after nutrition education within each group.

Lipid profiles	Conventional nutrition education group			Personalized nutrition education group			*P*
	Baseline	Follow-up	Change	Baseline	Follow-up	Change	
TC, mg/dL	172.71 ± 35.43	196.69 ± 41.74	23.98 ± 32.86	171.61 ± 35.55	200.78 ± 49.31	29.17 ± 41.85	0.05
HDL-C, mg/dL	46.71 ± 9.84	46.25 ± 10.43	-0.46 ± 8.67	46.51 ± 14.04	50.85 ± 11.35	4.33 ± 13.97	<0.01
LDL-C, mg/dL	102.59 ± 28.20	117.02 ± 28.94	14.43 ± 24.75	101.62 ± 25.91	116.75 ± 29.91	15.13 ± 24.27	0.81
TG, mg/dL	128.21 ± 105.47	153.28 ± 115.04	25.06 ± 107.00	136.61 ± 117.90	145.50 ± 157.13	8.89 ± 136.34	0.05
TC/HDL-C ratio	3.77 ± 0.71	4.39 ± 1.10	0.62 ± 0.85	3.78 ± 0.66	4.10 ± 1.32	0.32 ± 1.24	<0.01

TC, total cholesterol; HDL-C, high-density lipoprotein cholesterol; LDL-C, low-density lipoprotein cholesterol; TG, triglyceride.

Data are presented as mean ± SD.

p values were calculated by ANCOVA to compare the changes in serum lipid levels between groups, adjusting for age (years), sex (female and male), education level (primary school or below and junior high school or above), annual household per capita income (<5,000 yuan, 5,000-10,000 yuan, 10,000-20,000 yuan, ⩾20,000 yuan), marital status (married and others), body mass index (kg/m^2), smoking status (never, former, current smokers), alcohol drinking (never, former, current drinkers), physical activity (METs-hours/week), and total energy intake (kcal/day) at baseline.

Table S4. Changes in lipid profiles before and after nutrition education within each group among participants with dyslipidemias.

Lipid profiles	Conventional nutrition education group			Personalized nutrition education group			*P*
	Baseline	Follow-up	Change	Baseline	Follow-up	Change	
TC, mmol/L	4.71 ± 1.12	5.26 ± 1.26	0.56 ± 0.98	4.61 ± 1.1	5.36 ± 1.51	0.74 ± 1.26	0.03
HDL-C, mmol/L	1.14 ± 0.28	1.12 ± 0.26	-0.02 ± 0.23	1.16 ± 0.45	1.24 ± 0.29	0.08 ± 0.45	<0.01
LDL-C, mmol/L	2.76 ± 0.91	3.15 ± 0.87	0.38 ± 0.76	2.72 ± 0.79	3.16 ± 0.86	0.44 ± 0.68	0.28
TG, mmol/L	1.97 ± 1.49	2.16 ± 1.54	0.19 ± 1.54	1.97 ± 1.65	1.80 ± 1.63	-0.17 ± 1.17	0.01
TC/HDL-C ratio	4.17 ± 0.63	4.80 ± 1.11	0.64 ± 0.93	4.07 ± 0.62	4.44 ± 1.49	0.37 ± 1.52	0.04

TC, total cholesterol; HDL-C, high-density lipoprotein cholesterol; LDL-C, low-density lipoprotein cholesterol; TG, triglyceride.

Data are presented as mean ± SD.

p values were calculated by ANCOVA to compare the changes in serum lipid levels between groups, adjusting for age (years), sex (female and male), education level (primary school or below and junior high school or above), annual household per capita income (<5,000 yuan, 5,000-10,000 yuan, 10,000-20,000 yuan, ≥20,000 yuan), marital status (married and others), body mass index (kg/m^2), smoking status (never, former, current smokers), alcohol drinking (never, former, current drinkers), physical activity (METs-hours/week), and total energy intake (kcal/day) at baseline.

Table S5. Effects of personalized nutrition education on the serum profile changes compared with conventional nutrition education after exclusion of lipid-lowering medication users.

Lipid profile changes	β (95% CI)		*p*
	Conventional nutrition education group	Personalized nutrition education group	
TC, mmol/L	Reference	0.13 (0.00, 0.26)	0.06
HDL-C, mmol/L	Reference	0.12 (0.08, 0.16)	<0.01
LDL-C, mmol/L	Reference	0.01 (-0.08, 0.10)	0.8
TG, mmol/L	Reference	-0.20 (-0.38, -0.02)	0.03
TC/HDL-C ratio	Reference	-0.31 (-0.45, -0.16)	<0.01

TC, total cholesterol; HDL-C, high-density lipoprotein cholesterol; LDL-C, low-density lipoprotein cholesterol; TG, triglyceride.

p values were calculated in linear regression models to assess the difference of serum profile changes in the personalized nutrition education group compared with the conventional nutrition education group, adjusting for age (years), sex (female and male), education level (primary school or below and junior high school or above), annual household per capita income (<5,000 yuan, 5,000-10,000 yuan, 10,000-20,000 yuan, ≥20,000 yuan), marital status (married and others), BMI (kg/m^2), smoking status (never, former, current smokers), alcohol drinking (never, former, current drinkers), physical activity (METs-hours/week), and total energy intake (kcal/day) at baseline.

Table S6. Effects of personalized nutrition education on TG changes compared with conventional nutrition education in different subgroups.

Subgroup	N	β (95% CI)	$p_{interaction}$
**Sex**			0.44
Male	408	-0.31 (-0.65, 0.04)	
Female	510	-0.18 (-0.39, 0.02)	
**Age**			0.82
18-39	208	-0.18 (-0.54, 0.17)	
40-59	419	-0.29 (-0.61, 0.02)	
≥60	291	-0.07 (-0.36, 0.23)	
**Education level**			0.84
Primary school or below	508	-0.18 (-0.41, 0.05)	
Junior high school or above	410	-0.17 (-0.47, 0.13)	

TG, triglyceride.

$p_{interaction}$ values were calculated in linear regression models to assess the difference of HDL-C change in personalized nutrition education group compared with conventional nutrition education group, adjusting for age (years), sex (female and male), education level (primary school or below and junior high school or above), annual household per capita income (<5,000 Yuan, 5,000-10,000 yuan, 10,000-20,000 yuan, ≥20,000 yuan), marital status (married and others), BMI (kg/m^2), smoking status (never, former, current smokers), alcohol drinking (never, former, current drinkers), physical activity (METs-hours/week), and total energy intake (kcal/day) at baseline.

Table S7. Effects of personalized nutrition education on TC/HDL-C ratio changes compared with conventional nutrition education in different subgroups.

Subgroup	N	β (95% CI)	$p_{interaction}$
**Sex**			0.44
Male	408	-0.29 (-0.56, -0.02)	
Female	510	-0.33 (-0.46, -0.20)	
**Age**			0.41
18-39	208	-0.49 (-0.73, -0.25)	
40-59	419	-0.28 (-0.53, -0.04)	
≥60	291	-0.26 (-0.46, -0.05)	
**Education level**			0.45
Primary school or below	508	-0.27 (-0.42, -0.12)	
Junior high school or above	410	-0.36 (-0.61, -0.11)	

TC, total cholesterol; HDL-C, high-density lipoprotein cholesterol.

$p_{interaction}$ values were calculated in linear regression models to assess the difference of HDL-C change in the personalized nutrition education group compared with the conventional nutrition education group, adjusting for age (years), sex (female and male), education level (primary school or below and junior high school or above), annual household per capita income (<5,000 yuan, 5,000-10,000 yuan, 10,000-20,000 yuan, ≥20,000 yuan), marital status (married and others), BMI (kg/m^2), smoking status (never, former, current smokers), alcohol drinking (never, former, current drinkers), physical activity (METs-hours/week), and total energy intake (kcal/day) at baseline.
